# Quantifying Socioeconomic and Lifestyle Related Health Risks: Burden of Cardiovascular Disease Among Indian Males

**DOI:** 10.5195/cajgh.2015.218

**Published:** 2015-12-22

**Authors:** Neetu Purohit, Divya K. Bhati, Shiv D. Gupta, Azad S. Kundu

**Affiliations:** 1 Indian Institute of Health Management Research University;; 2 Indian Council of Medical Research

**Keywords:** cardiovascular diseases, risk factors, lifestyle, behavior, smoking, physical activity

## Abstract

**Background:**

Non-communicable diseases account for a significant disease burden in the South East Asia region. India is facing an increased incidence of lifestyle-related diseases, such as cardiovascular disease. Socioeconomic and lifestyle risk factors for cardiovascular disease (CVD) have been under investigated in India. This study was designed to explore risk factors contributing to the development of cardiovascular disease among Indian males.

**Methods:**

A population-based cross-sectional study was conducted among 2,235 males in the age group of 18–60 years across three states of India. A household survey was used to collect demographic and socioeconomic status information in addition to lifestyle-related attributes such as smoking, alcohol consumption, diet, and physical activity. Descriptive statistics and logistic regression were performed to identify the role of various factors that may be associated with the development of cardiovascular disease in this population.

**Results:**

The prevalence of cardiovascular disease among the male respondents contacted through a household survey was reported to be 9.8%. Logistic regression revealed that males with higher education and higher income were more likely to report CVD. With age as a strong predictor of CVD, the risk of CVD was found to be five times higher in the older age group. Current smokers were 1.3 times more likely to have CVD compared to those who never smoked. Those who were engaged in physical activity were less likely to have CVD; however, the adverse effects of smoking and excessive consumption of red meat showed a stronger association with CVD than the protective effects of physical activity.

**Conclusion:**

In developing countries, where the increase in earning capacity and change in lifestyle has been found to be accompanied by substantial risk of heart disease for males, public health measures like health promotion programs need to be implemented to decrease CVD burden.

Non-communicable diseases (NCDs) account for an increasing share of disease burden. In 1998, 43% (36 million) of global mortalities were due to NCDs and are expected to increase to 73% by 2020.^1^ Among NCDs, cancer, cardiovascular disease (CVD), and diabetes are of serious concern, accounting for 52% of all deaths and 38% of disease burden in the World Health Organization South East Asia Region (WHO-SEAR). In the industrialized world and developing nations such as India,^2^ CVDs are the primary causes of death and disability.^3^ According to WHO, an estimated 17 million people died from CVD in 2005, comprising 80% of the deaths in low and middle income countries. In a developing country like India, 35% of all CVD deaths occur in working age adults (aged 35–64 years) which makes prevention of these deaths crucial for society.^4^

Unlike other non-communicable diseases, CVD is one of the most preventable causes of death in the world, as a majority of its risk factors are controllable. Worldwide, approximately 31% of heart disease is believed to be attributable to poor diet and 22% to physical inactivity, and a further 22% is estimated to be attributable to smoking in industrialized countries.^5^ Until the 1990s, CVD was regarded as a disease of the affluent class in India.^6^ However, studies conducted in 1996, 2004, and 2006 showed a higher incidence of CVD events among lower socioeconomic groups, reflecting a reversal of the social gradient of CVD within socially disadvantaged groups in India.^7^–^10^

There are significant gaps in the knowledge of CVD associated risk factors in countries of the South Asian region. In India, there has been no national study that has used a uniform methodology to assess prevalence of multiple cardiovascular risk factors. Existing studies in India were done at different geographic locations and in different time periods.^11^ A recently published report indicated that new and more rigorous research and surveillance studies are needed in India to assess the extent of NCD burden.^12^ Due to scarce national level data, there is a lack of effort to initiate policy changes for controlling the CVD epidemic.^13^ However, a few public health sentinel surveys have found that a cluster of major risk factors (tobacco, alcohol, inappropriate diet, and physical inactivity) govern the occurrence of CVDs in India.^14^ Studies have also found that rapid dietary changes associated with a decrease in levels of physical activity also play a particularly important role in the increasing incidence of cardiovascular diseases.^15^

The purpose of this multi-site study was to determine the contribution of several socioeconomic and lifestyle factors to cardiovascular diseases among the adult male population of India. It was hypothesized that lifestyle factors, such as socioeconomic characteristics (e.g. age, education, place of residence, income, occupation) and lifestyle factors (e.g. smoking, alcohol consumption, red meat consumption, physical activity) are predictors of CVD in Indian population.

## Methods

### Sample size calculation, site, and study tools

**Figure 1.**
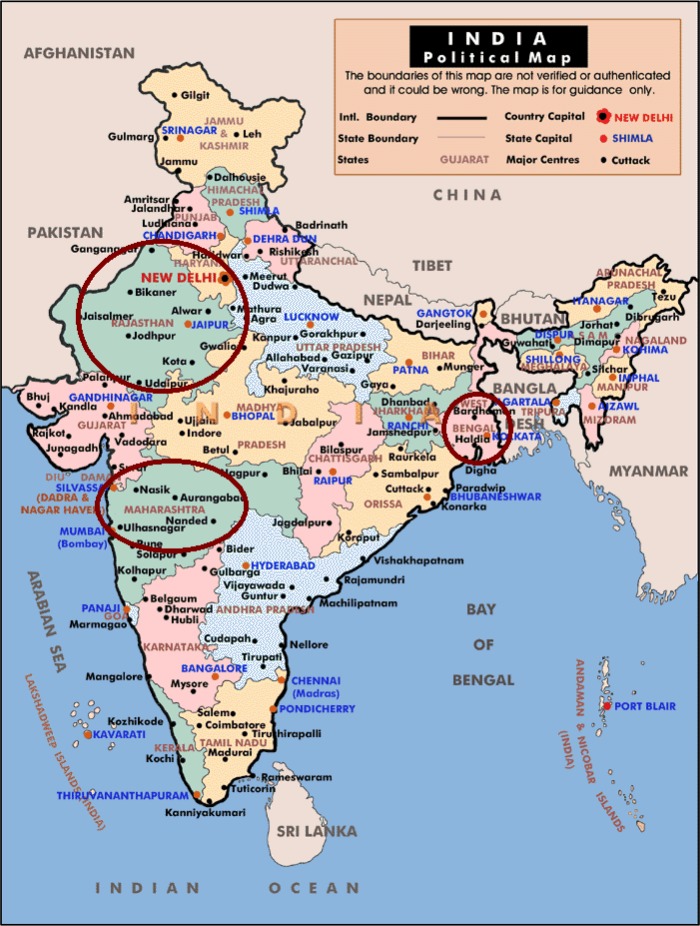
**Study locations in India: Rajasthan, Maharashtra and West Bengal**

A population-based, multi-centric, cross-sectional survey was conducted in three major states in India, including Rajasthan, Maharashtra, and West Bengal, which represent the Northern, Western, and Eastern regions of India, respectively (Figure 1). The appropriate sample size needed was determined using the standard formula as described in World Health Organization sample size determination manual.^16^

**Figure 2.**
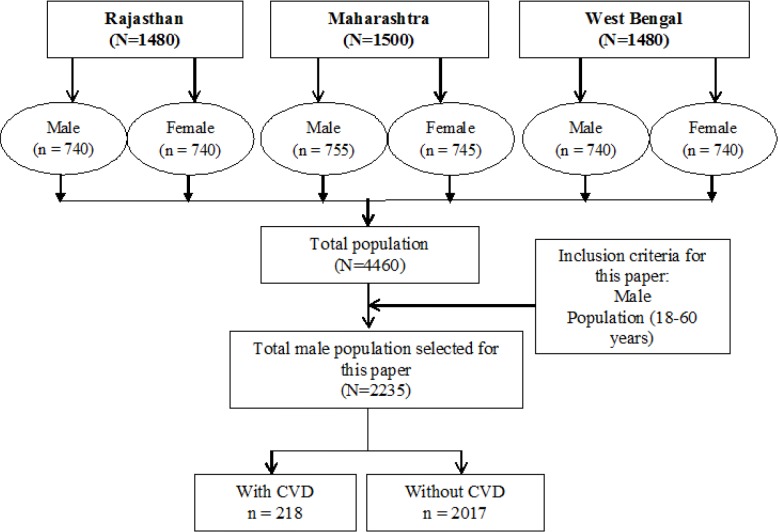
**Sample selection flow-chart**

The sample size of 1,480 adults per state was calculated using 10% precision and took into account a non-response rate of 15%. Thus, the total study sample included 4,460 adults in the age group of 18–60 years (2,225 females and 2,235 males). This paper presents the sub-study focusing on data collected from 2,235 male participants (Figure 2). In 2001–2003, a large scale study was conducted by the Registrar General of India, which reported that cardiovascular disease (CVD) was the major cause of deaths in males (20.3%) as compared to females (16.9%);^17^ hence, the authors decided to report results on the male population first. Further, the data from females was not considered for this particular paper as the risk factors for females are not necessarily identical to males and a separate analysis quantifying risk factors among females is proposed in a separate follow up publication.

The multilingual household survey questionnaire was prepared by the Indian Council of Medical Research (ICMR, Delhi). The structured survey questionnaire and its contents were validated with the help of literature review and face validity was conducted with experts working in the area of NCDs / CVDs. The questionnaire was translated into Hindi / relevant regional languages and subjected to forward and backward translation. The structured questionnaire was pre-tested with respondents with similar characteristics who reside in a different geographical area (other than the study area) to ensure the sequencing and understanding of the questions. Suggestions from the study team and experts were incorporated into the questionnaire before its administration within the study area. Ethical clearance for the study was obtained from the ethical review board of Indian Council of Medical Research (ICMR), which was the funding agency for this multi-centric study. Verbal consent (due to concern about literacy level) was obtained from study respondents prior to survey administration. The study team was comprised of a principal coordinator and research officers. The study team provided uniform study procedure-related training to the field investigators of the respective states. The field investigators administered the structured questionnaire through face-to-face interviews. The responses were recorded by the field investigators in a standardized format.

### Definitions and measurement

The outcome variable was the presence or absence of self- reported cardiovascular disease, which was defined as per World Health Organization.^17^ For the purpose of this study, cardiovascular disease has been defined as coronary heart disease (heart attacks), cerebrovascular disease (stroke), high blood pressure (hypertension), peripheral artery disease, rheumatic heart disease, congenital heart disease, and congestive heart failure.^18^ Information on demographic, socioeconomic and lifestyle risk factors was ascertained through the questionnaire developed by the study team. Study population was divided into two age categories (18 to 40 years) and (41 to 60 years). Current place of residence was classified as rural or urban. Self-reported educational attainment levels were grouped in five categories (i.e. illiterate/no-schooling, primary [≤5^th^grade], secondary [≤12^th^ grade], graduate [including post-graduate], and professional degree). Occupation was divided into 8 categories: skilled or unskilled labor, agriculture, military/police, office job, business/shop, managerial position, unemployed, and teachers/small traders. Per capita monthly family income from all sources was grouped into three categories: low income group (LIG): < 5,326 INR (< $83.75), middle income group (MIG): 5,327 INR – 21,067 INR ($83.76 – $331.25), and high income group (HIG): > 21,068 INR (>$331.26).^19^ As per the Global Adult Tobacco Survey report in 2009,^20^ current smoker has been defined as a person who had smoked over their lifetime, and continued to smoke every day or some days. Ever smoker was defined as a person who had smoked sometime in their lifetime, but does not smoke currently. A never smoker was defined as a person who never smoked over their lifetime.^20^ Current alcohol user was defined as a person who consumes alcohol every day or some days. Ever alcohol user was defined as a person who was consuming alcohol in the past and stopped consuming alcohol. A never alcohol user was defined as person who never consumed alcohol over their lifetime.^21^ Diet was categorized in terms of consumption of non-vegetarian food (i.e. consumption of red meat daily or 3 to 5 times a week, and no consumption of red meat). Physical activity time was defined based on the WHO global recommendation on involvement in any physical activity for 30 minutes or more per day.^22^

### Statistical analysis

Descriptive statistics were used to describe the sample characteristics. Bivariate regression model estimates the contribution of indicators of socioeconomic status and lifestyle factors to self reported CVD. The dependent variable was presence or absence of self-reported CVDs. Statistical assessments were carried out using Statistical Package for Social Sciences (SPSS) version 20.0 (Chicago IL). All statistical analyses were considered significant when the *p*-value was < 0.05 at 95% confidence interval.

## Results

### Population characteristic: Socioeconomic and lifestyle attributesof respondents

A total of 2,235 males from three states were included in the present subanalysis. Their socioeconomic status and lifestyle factors are presented in Table 2 by geographical region. Of the total interviewed males, the prevalence of self-reported cardiovascular disease was found to be around 9.8%. The age of interviewed respondents ranged from 18 to 60 years with a mean age 37.51+12.16 (Table 2). The majority (61.0%) of respondents were in the 18 to 40 years age group.

Table 1 also shows that approximately 45% of the respondents were residing in rural areas. Nearly 37.5% of the respondents completed elementary education. Education beyond grade school was reported by 31.3% males and 10.9% reported having a professional degree. Occupation of the interviewed respondents shows that around 40.6% were engaged in professions involving physical activity, such as skilled /unskilled labor, agriculture, and police or military service. About 21% and 12.9 % of the respondents were from high and middle income categories, respectively. The proportion of smokers, alcohol consumers, those who consumed red meat daily (or 3 to 5 times a week), and those engaged in regular physical activity was also assessed. Results showed that never smokers and those who never consumed alcohol constituted 50.0% and 64.1% of the sample, respectively. About 18.1% of the respondents consumed red meat daily or 3 to 5 times a week. Results also showed that involvement in regular physical activity was as high as 76.0%.

### Predictors of CVD

Based on bivariate logistic regression analysis, Table 2 illustrates the estimated odds ratios for association between CVD and socioeconomic and lifestyle related attributes. Age was a strong predictor of CVD; males who were 41–60 years old had a 5-fold increased risk of having CVD compared to those in the age group of 18–40 years. Compared to males who were illiterate or have received only non-formal education (i.e. able to write their names and read and write on rudimentary level), those with a professional degree were 2.1 times more likely to have CVD. Further, males who were living in urban areas were 1.5 times more likely to have CVD as compared to those living in rural areas.

For occupation, compared to those engaged in skilled/unskilled labor, males in teaching and trading jobs were 1.8 times more likely to get CVD. The odds for other occupation categories did not show a significant relationship with CVD. Income level has also been associated with CVD level. Compared to those in a low income category, participants with a high income had a marginally significant increased odds of having CVD (OR=1.39, *p*=0.09). With respect to lifestyle related attributes, smoking and consumption of red meat daily or 3–5 times a week showed a significant positive relationship with CVD. Males who consumed red meat were 1.6 times more likely to have CVD than those who did not consume red meat at all. Smoking was found to be adversely affecting cardiovascular health. Compared to those who did not smoke, smokers were 1.3 times more likely to get CVD. Engagement in physical activity showed a negative significant relationship with CVD, which indicated that compared to those who did not engage in physical activity, those who engaged in physical activity had 0.7 times less likelihood of reporting CVD.

Encouraged by the findings on the protective role of physical activity for CVD, we attempted to investigate the interaction effect of physical activity with red meat consumption and physical activity with smoking. These lifestyle variables were univariably marginally or significantly associated with CVD. We then tested whether or not there was an interaction between the lifestyle factors; however, all interactions tests were non-significant (Table 3).

The first interaction examined was the interaction between red meat consumption and physical activity. Red meat consumption increased the odds of having CVD, although not significant, while physical activity significantly reduced the odds of reporting CVD. The interaction effect suggested that there may be an interaction between these two factors, as the significance of red meat consumption was attenuated when adjusting for this interaction. However, this interaction effect was not found to be statistically significant (*p*=0.40). Our results suggest that the risks of CVD associated with excessive consumption of red meat cannot be ruled out, even in presence of engaging in physical activity. The second interaction examined was the one between smoking and physical activity. Simlarly with the previous interaction test, physical activity was still significantly associated with lower odds of having CVD, while smoking increased the odds of reporting CVD. This interaction effect also suggests that there may have been an interaction between smoking and physical activity due to the attenuated significance of the effects of smoking. However, this interaction was not found to be statistically significant (*p*=0.33).

## Discussion

This study examined the association between lifestyle and CVD among Indian males. Physical activity was found to reduce the odds of having CVD by 28%, while red meat consumption and smoking increased the odds of having CVD by 62% and 38%, respectively.

Major causes of morbidity and mortality have undergone an epidemiologic transition from predominantly nutritional deficiencies and infectious diseases in developing nations to chronic diseases such as cardiovascular disease.^23^ With the epidemiological transition and increased urbanization associated with the increase in CVD risk factors (behavioral, social, and lifestyle patterns), the CVD burden is increasing in developing countries including India.^24^ Previous literature provides evidence that while the prevalence and mortality rate of CVD has decreased in developed nations, it has substantially increased in India, which has been undergoing rapid demographic, social, and economic change.^25^ Studies have shown a major income decline due to CVD-related losses in productive labor.^3^ Understanding the health characteristics of young and middle aged males (18–60 years) will allow public health experts and policy makers to adopt focused interventions for prevention of disease and promoting lifestyle modifications.

Age, as a risk factor for CVD, is well established^26^ and was confirmed in the current study. Prevalence of cardiovascular disease was found to be higher in the age group of 41–60 years. Additionally, males with higher education were found to be more vulnerable to the risk of reporting cardiovascular disease, which could be due to bias, as more educated adults may be more likely to report a disease. An earlier study carried out in the capital city of India also demonstrated that prevalence of heart disease was higher among literate as compared to illiterate participants.^26^ Thus, population-specific interventions need to be designed for various educational groups to promote a healthy lifestyle.

The study findings provided further evidence suggesting that males in the high income group had an increased likelihood of reporting CVD. A study from southern India also reported a higher prevalence of coronary heart disease in high socioeconomic groups.^27^ Occupation is also used as a marker in epidemiological studies of cardiovascular disease. A systematic review carried out in 2010 demonstrated an increased risk of cardiovascular disease in occupations involving prolonged sitting.^28^ However, the current study did not find any significant association of CVD with office jobs. We found that teachers and small traders are more vulnerable to CVD, but this finding was only marginally significant. The current study also found that urban males were at significantly greater risk compared to their rural counterparts. This is consistent with other published studies.^29^,^30^

Corroborating earlier studies, this study reported that smoking adversely affects cardiovascular health. Smoking has been shown to be a major risk factor for chronic illnesses in males below 65 years of age in developing countries.^31^ In long-term smokers, smoking is responsible for more than 50% of avoidable deaths, and one half of these are due to CVD.^31^ Our study did not establish any significant association of alcohol consumption with CVD. However, the results of current study support the findings of 2010, WHO-Global status report on Non-Communicable Diseases, which states that there is a direct relationship between higher levels of alcohol consumption and the rise in risk of cardiovascular disease, which is dependent on the amount and pattern of alcohol consumption.^32^

A systematic review carried out between 1992 and 2009, which included 17 cohort and 3 case-control studies, showed that processed red meat was associated with a higher incidence of coronary heart disease (CHD) when compared with unprocessed red meat.^33^ A previously published study failed to establish a significant link between meat consumption with chronic diseases like coronary heart disease (CHD), stroke, and Type 2 diabetes mellitus.^34^ Thus, this study provides interesting new information.

The benefits of physical activity on cardiovascular health were well established in the past review of key issues in public health.^35^ People who are insufficiently physically active have a 20–30% increased risk of all-cause mortality compared to those who engage in at least 30 minutes of moderate intensity physical activity on most days of the week. Previous studies also suggest that physical activity lowers the risk of stroke and hypertension.^36^ Consistent with previously published research, this study also reported the importance of regular physical activity in reducing the risk of CVD. This study reinforces the need to consider current prevention and treatment strategies at the national level to include behavior change,^37^ early diagnosis, screening, and early detection. Cardiovascular health awareness programs including but not limited to those focusing on obesity and smoking need to be continued and expanded to better educate populations about healthy lifestyles. However, awareness is only the first step towards behavior change and other factors, like developing public health infrastructure (i.e. parks, cycling tracks, public sport centers, primary care clinics, obesity prevention programs, addiction treatment centers), that could facilitate healthy lifestyle choices to be promoted.

The results from this study add to the existing knowledge on the associated risk factors and their role in causing cardiovascular diseases among males in India. The primary limitation of this study was that no medical examination was conducted to assess the presence of cardiovascular diseases, and only self-reported cardiovascular diseases were taken into consideration. However, the findings of this study are relevant for other developing countries undergoing the edpidemiologic transition. Public health programs need to focus on building awaremenss about healthy lifestyles and changing public health infrastructure to promote healthier lifestyles, which could benefit all segments of society.

## Figures and Tables

**Table 1.** Percent distribution of respondents by socioeconomic characteristics and lifestyle factors

Variables	Northern Region M±SD or %	Western Region M±SD or %	Eastern Region M±SD or %	Total M±SD or %
N	740	755	740	2235
Age	38.45±11.75	36.64±12.93	37.46±11.69	37.51±12.16
Age group in years				
18–40	59.9	62.6	60.4	61.0
41–60	40.1	37.4	39.6	39.0
Cardiovascular disease status				
No	91.2	93.5	85.9	90.2
Yes	8.8	6.5	14.1	9.8
Area of residence				
Rural	40.5	53.4	40.4	44.8
Urban	59.5	46.6	59.6	55.2
Education level				
Primary	35.9	29.3	47.6	37.5
Secondary	33.9	37.0	23.0	31.3
Graduate	16.5	24.8	19.5	20.3
Professional	13.6	9.0	10.0	10.9
Occupation				
Skilled/unskilled labour	26.1	20.5	31.8	26.1
Agriculture	14.3	17.1	9.1	13.5
Official/job-clerical	8.2	20.8	15.1	14.8
Military/police	0.7	1.3	0.4	0.8
Business/shops	26.2	15.2	24.7	22.0
Manager/professional	8.5	10.3	6.2	8.4
Unemployed	4.5	8.2	3.8	5.5
Teacher/small traders	11.5	6.5	8.9	8.9
Income				
Low income	100.0	44.6	53.9	66.0
Middle income	0	22.8	15.8	12.9
High income	0	32.6	30.3	21.0
Smoking status				
Current/ever smoked	51.2	36.8	62.2	50.0
Never smoked	48.8	63.2	37.8	50.0
Alcohol consumption				
Current/ever use	33.0	30.9	43.9	35.9
Never use	67.0	69.1	56.1	64.1
Red-meat consumption				
No consumption	94.2	89.9	61.5	81.9
≥3 times/week	5.8	10.1	38.5	18.1
Engagement in physical activity				
0 to 30 minutes	24.5	15.8	32.3	24.0
>30 minutes	75.6	84.2	67.7	76.0

Note: M: Mean, SD: Standard Deviation

**Table 2.** Bivariate logistic regression analysis of predictors of cardiovascular diseases

Risk factors	Odds ratio	95% CI	*p*-value
Age in years			
18–40	-	-	-
41–60	4.57	3.29 – 6.34	<0.001**
Area of residence			
Rural	-	-	-
Urban	1.59	1.09 – 2.29	0.014*
Education			
Primary	-	-	-
Secondary	1.25	0.78 – 1.98	0.354
Graduate	1.66	0.97 – 2.82	0.064†
Professional	2.11	1.03 – 4.33	0.041*
Occupation			
Skilled/unskilled labour	-	-	-
Agriculture	1.03	0.54 – 1.95	0.924
Official/job-clerical	1.56	0.85 – 2.82	0.146
Military/police	1.36	0.26 – 6.90	0.712
Business/shops	1.37	0.80 – 2.33	0.249
Manager/professional	1.24	0.62 – 2.47	0.542
Unemployed	1.38	0.58 – 3.22	0.463
Teachers/small traders	1.85	0.98 – 3.46	0.054†
Income			
Low income	-	-	-
Middle income	0.76	0.44 – 1.29	0.310
High income	1.39	0.94 – 2.02	0.092†
Smoking status			
Never smoked	-	-	-
Current/ever smoked	1.383	0.99 – 1.91	0.052†
Alcohol consumption			
Never use	-	-	-
Current/ever use	1.17	0.84 – 1.61	0.333
Red meat consumption			
No consumption	-	-	-
≥3 times/week	1.615	1.125 – 2.31	0.009**
Engagement in physical activity			
0 to 30 minutes	-	-	-
30 minutes or more	0.719	0.51 – 1.00	0.052†

*Note*:

† *p*< 0.1,

* *p*< 0.05,

** *p*< 0.001

**Table 3.** Logistic regression of predictors of cardiovascular diseases: Examining interactions

	Odds Ratio	95% CI	*p*-value
Physical activity (≥30 minutes)	0.39	0.25 – 0.62	<0.001**
Red meat consumption (≥3 times/week)	1.43	0.89 – 2.29	0.138
Physical activity × Red meat consumption	1.32	0.68 – 2.56	0.409

Physical activity (≥30 minutes)	0.39	0.25 – 0.62	<0.001**
Smoking status (current/ever)	1.37	0.94 – 1.99	0.103
Physical activity × Smoking status	1.33	0.74 – 2.39	0.335

*Note*:

† *p*< 0.1,

* *p*< 0.05,

** *p*< 0.001
